# Kinesiophobia – Introducing a New Diagnostic Tool

**DOI:** 10.2478/v10078-011-0019-8

**Published:** 2011-07-04

**Authors:** Andrzej Knapik, Edward Saulicz, Rafał Gnat

**Affiliations:** 1 Department of Health Care, Silesian Medical University, Katowice, Poland; 2 Department of Physiotherapy, Academy of Physical Education, Katowice, Poland; 3 Department of Physiotherapy, Academy of Physical Education, Katowice, Poland

**Keywords:** low motor activity, hypokinesia, fear of movement

## Abstract

Technical development of human civilisation brings about a decrease of adaptation potential of an individual, which is directly linked to deficient motor activity. Only precise identification of factors leading to hypokinesia would make prophylactic and therapeutic actions possible. In this article, authors would like to introduce a new, original tool aiming at diagnosing limitations of motor activity in adults. They propose a synthetic diagnosis of hypokinesia in two domains: biological and psycho-social, which is based on the contemporary model of health.

## Introduction

Technical development, besides its unquestionable advantages, brings about numerous threats. From the perspective of human health, the greatest risk consists of decreasing adaptation potential of an individual. This unfavourable change constitutes a consequence of limited environmental demands for motor activity, including both locomotion, as well as an abundant range of various motor patterns providing a base for material existence. From a prophylactic perspective, maintaining an optimal (possibly high) level of adaptation potential requires an equally high level of motor activity. Most often, such forms of activity are used which reflect different forms or components of daily life activities and aim to either maintain or increase fitness of the organism, i.e. physical exercises (Caspersen et al., 1985). Studies in this topic usually search for direct links between levels and types of activity and certain physiologic and morphologic parameters as well as consequences of activity from psychological, social and economic perspectives (Haskell & Wolffe, 1994; Wolinsky et al., 1995; Bouchard et al., 1998; 1999; Batty, 2002; Vainio & Bianchini, 2004; La Fontaine, 2008; McNeely & Courneya, 2010; Diep et al., 2010). New standards, very valuable from a population’s point of view, are constantly being developed (Pate et al., 1995; Blair et al., 2004; Kushi et al., 2006; AHS, 2007; WHO, 2010). However, to commence application of any exercise, a proper diagnosis of the activity level and existing limitations is necessary.

Literature provides a great deal of information on determinants of motor activity. Several of them were already described: demographic and biological, psychological, cognitive, emotional, social, cultural and others linked to components of a given motor ability (Sallis & Owen, 1999). Authors also tend to emphasise the role of social support in attempts to increase motor activity (Bandura, 1986; Martin-Matillas, 2010). Influence of gender (Pate et al., 1994), genetic factors (Pérusse et al., 1989) and individual commencement have been mentioned (Mc Auley & Blissmer, 2000). The importance of psycho-social factors, including the whole complexity of human behaviour, cannot be overestimated (Zhang & Middlestadt, 2007). All this may create an impression that difficulties accompanying exploration of the described domain are impossible to be overcome. However, the increasing number of research providing evidence on prophylactic and therapeutic effects of physical activity prove the opposite. Finally, every consideration leads to one fundamental question: Why do many people prefer passive lifestyle although they are aware of numerous advantages of motor activity (La Fontanie, 2008; Drygas et al., 2008; Martin, 2010)? To answer such a question, one must understand the meaning of limitations of the activity. Great variability of human behaviour, as well as numerous factors influencing the level of activity prompt to approve a simplified theoretical model explaining the reasons for commencing motor activity, in which the broad spectrum of motivations and limitations is reduced to two major items: (1) biological: structural, morphologic, energetic, instinctive; (2) psycho-social: personality, culture, emotions. It is worth mentioning that practically the border between the two mentioned spheres is somehow blurred and it is frequently the case that the most important limitation of motor activity is fear of movement, regarded as a component of personality of an individual. This type of attitude is referred to in literature as kinetophobia or kinesiophobia. Kori et al. (1990) defined kinesiophobia as irrational, weakening and devastating fear of movement and activity stemming from the belief of fragility and susceptibility to injury. These authors proposed a questionnaire aiming to diagnose kinesiophobia: The Tampa Scale of Kinesiophobia. It is mainly addressed to adult either acute or chronic low back pain patients. However, it may also be successfully used in neck pain patients or individuals suffering from post-traumatic musculo-skeletal ailments. The Tampa Scale constitutes therefore a psychometric, clinically-oriented diagnostic, prognostic and monitoring tool (Woby et al., 2005, Houben et al., 2005, Roelofs et al., 2007). Contemporary, the phenomenon of kinesiophobia has however a broader range of influence and cannot be fully explained as simple fear of pain. It may well appear as a fear of physiological symptoms of fatigue or exhaustion or, even more comprehensively, fear of physical or mental discomfort. Taking into account biological determinants of motor activity, it may be assumed that motor passivity, regarded as a dissonance between true possibilities and demands of an individual and internal picture of his/her motor potential, is also a symptom of kinesiophobia. In turn, this internal imprint of motor potential is surely shaped by social influence.

Generally, all fear behaviours are rooted in compromised feeling of safety. In case of kinesiophobia, various defence mechanisms may appear, such as: repression (removing from consciousness), negation (there is no need for movement), simulation and projection (sports fan behaviour) or, most frequently used, rationalisation (e.g. lacking time). Typical psychosomatic symptoms are rather rare and may only appear when kinesiophobic individual is, by any means, forced to increase activity.

Prophylactic issues prompt to treat kinesiophobia rather as a feature of an individual personality (constant or temporary) than symptoms of mental disturbance. A category of “avoidance behaviour” seems to be most suitable in this case. The need for proper diagnosis of the causes and intensity of kinesiophobia is evident.

## Objective

In this article, authors would like to introduce a new, original tool aiming to diagnose limitations of motor activity. These limitations must be described in the light of phobic behaviours, which normally are not susceptible to change, of a relatively constant intensity, connected with avoidance of triggering factors and irrational. According to the lifestyle definition, in case of kinesiophobia, the lacking susceptibility to change would place given individual in the category of passive people. However, this is not sufficient since there is also a need to evaluate intensity of kinesiophobic attitudes. Therefore, a numeric scale is proposed in which a score of 100 would mean highly kinesiophobic attitude (clinical form of fear of movement) and 0 would represent lack of any symptoms of kinesiophobia. The criterion of irrationality, yet debatable, might be considered as ignoring medical indications regarding motor activity. New standards addressing optimal physical loads in populations of either healthy individuals or patients are constantly being developed. However, these tend to be ignored, which indicates a type of phobic attitude suggesting a need for professional psychotherapeutic intervention.

Taking into consideration all remarks mentioned above, a new diagnostic tool was developed and is proposed in this article, named: Kinesiophobia Causes Scale (KCS). It is devised for an adult population and aims to diagnose original causes of motor passivity.

### Identification of causes of kinesiophobia

Our paradigm is a holistic definition of health, including its physical and mental dimension, both influenced by social factors. The proposed scale is therefore divided in two domains: biological and psychological. Biological domain includes the following causes of kinesiophobia:

- morphologic
- individual need for stimulation
- energetic substrates
- power of biological drives.

Psychological domain contains:

- self-acceptance
- self-assessment of motor predispositions
- state of mind
- susceptibility to social influence.

Such a construct allows to diagnose individual causes of kinesiophobia and their intensity in the two domains separately, as well as to calculate the total score of KCS.

### Kinesiophobia Causes Scale

***Dear Madam/Dear Sir You are kindly requested to choose ONE answer for EACH item below except items 8 and 13 where you will choose between yes/no/not sure for EACH answer***

**1. According to my body mass, I can claim that:**

**Table:** 

a) I control my body mass adjusting the level of motor activity	**[0]**
b) my body mass causes difficulties in performing several motor tasks, so I avoid them	**[50]**
c) due to overweight, I avoid physical efforts because of the risk of exhaustion or injury	**[100]**

**2. I feel that because of its shape, my body causes motor limitations in sport activities I would like to perform:**

**Table:** 

a) almost never	**[0]**
b) seldom	**[25]**
c) sometimes	**[50]**
d) often	**[75]**
e) very often	**[100]**

**3. I think that in comparison with others I am always perceived as:**

**Table:** 

a) less active	**[100]**
b) less active than people of my age	**[75]**
c) equally active as people of my age	**[50]**
d) more active than other people	**[25]**
e) far more active than other people	**[0]**

**4. Prolonged sitting:**

**Table:** 

a) feels pleasant to me, I can assume and maintain such position for a long time	**[100]**
b) similarly to other people, when it lasts too long, I have to change position	**[50]**
c) seems uncomfortable to me, I avoid prolonged sitting	**[0]**

**5. While working, I try to find a way demanding the least physical effort because I do not like physical fatigue:**

**Table:** 

a) always	**[100]**
b) sometimes	**[50]**
c) never	**[0]**

**6. I believe that activities demanding intensive physical effort:**

**Table:** 

a) are fatiguing to me and I try to avoid them if possible	**[100]**
b) are possible, it depends what specifically I should do	**[50]**
c) give me pleasure because physical fatigue means satisfaction to me	**[0]**

**7. When I am physically exhausted:**

**Table:** 

a) I feel bad and it takes long time to recover	**[100]**
b) I recover as quickly as other people of my age	**[50]**
c) I recover quickly and I feel energy to start new actions	**[0]**

**8. I believe that irrespectively of my present state of mind I could with** **NO rest:**

**Table:** 

	**yes**	**not sure**	**no**
a) walk for 1 hour	**[0]**	**[50]**	**[50]**
b) climb third floor	**[0]**	**[100]**	**[100]**
c) ride a bike for 0.5 hour	**[0]**	**[50]**	**[100]**

**9. After work, I usually feel:**

**Table:** 

a) tired, but after a little rest, I am ready to start activity (housework, visiting friends, going to the cinema, theatre, walking or sport)	**[0]**
b) tired and I rest passively either lying or sitting	**[50]**
c) rather exhausted than tired, and I always rest for a long time either lying or sitting	**[100]**

**10. Competition in sport, work, etc.:**

**Table:** 

a) always makes me satisfied and gives opportunity to win	**[0]**
b) is acceptable in disciplines I feel good at, then I like to compete	**[50]**
c) is out of question, I’m very sensitive to failures	**[100]**

**11. I feel irritated when circumstances force me to park a car far from destination:**

**Table:** 

a) always	**[100]**
b) often	**[75]**
c) sometimes	**[50]**
d) seldom	**[25]**
e) never	**[0]**

**12. In relation to my own appearance:**

**Table:** 

a) I never felt embarrassed by the shape of my body. Wearing clothes exposing it (e.g. sport clothes or) swimsuit do not seem problematic to me irrespective of how do other people look like	**[0]**
b) I can wear sport or swimsuit on the condition that people around look similarly	**[50]**
c) I avoid situations in which clothing would expose shortcomings of my figure	**[100]**

**13. I believe that activities mentioned below should, because of cultural reasons, match age and/or social status of a given individual:**

**Table:** 

	**yes**	**no**
a) dancing	**[100]**	**[0]**
b) sport	**[100]**	**[0]**
c) fatiguing non-profit tasks(e.g. housework, gardening, DIY)	**[100]**	**[0]**

**14. At the opportunity of participation in sport (holidays, encouragement from other people):**

**Table:** 

a) I always try to use it	**[100]**
b) I feel certain resistance, but usually I agree	**[75]**
c) first I watch others and try to judge my chances for good performance and then I take my decisions	**[50]**
d) it is very difficult to convince me, I rarely agree	**[25]**
e) no, this is not for me	**[0]**

**15. In comparison with other people, I believe that I can learn new movements (motor skills):**

**Table:** 

a) more quickly than others	**[0]**
b) more quickly than people of my age	**[25]**
c) as quickly as people of my age	**[50]**
d) more slowly than others	**[75]**
e) I cannot learn any motor skill	**[100]**

**16. During my childhood and adolescence:**

**Table:** 

a) I did not participate in sport (only obligatory exercises)	**[100]**
b) I participated in sport as often as other kids	**[50]**
c) I was more active than others (e.g. training in a sport club)	**[0]**

**17. Considering pain, trauma and injuries:**

**Table:** 

a) I believe that in life, there is always a risk of sickness and injury, but this is not a factor reducing my motor activity	**[0]**
b) I believe that it is necessary to act in accordance to the “common sense” and adjust the level and type of activity to an individual’s age and abilities	**[50]**
c) I believe that increased activity may be harmful, special care should be taken	**[100]**

**18. When I become sick or sustain an injury, I believe that:**

**Table:** 

a) first is to recover completely and then to start regular activity	**[100]**
b) reasonable level of motor activity is necessary, in accordance to medical indications and my own condition	**[50]**
c) frequently the best way to fight the problem is to ignore the pain and lead normal, active life	**[0]**

**19. In comparison with my relatives, friends and mates:**

**Table:** 

a) I rest more actively than they do	**[0]**
b) I rest typically for my age and gender	**[50]**
c) I rest more passively than they do	**[100]**

**20. In comparison with other expenses, expenses on active recreation are for me:**

**Table:** 

a) less important	**[100]**
b) equally important	**[50]**
c) more important	**[0]**

### Calculation of KCS scores

According to our assumptions scores obtained for the biological and psychological domains, as well as the total KCS score will range from 0 to 100 and can be interpreted as percent of kinesiophobic behaviour. Calculations for items 8 and 13 are performed as follows:

Item 8=**(a+b+c)/3**
Item 13=**(a+b+c)/3**

Calculations for individual causes of kinesiophobia are performed as follows:

- morphologic**=items(1+2)/2**
- individual need for stimulation**=items(3+4+5)/3**
- energetic substrates**=items(6+7+8+9)/4**
- power of biological drives**=items(10+11)/2**
- self-acceptance**=items(12+13+14)/3**
- self-assessment of motor predispositions **=items(15+16)/2**
- state of mind**=items(17+18)/2**
- susceptibility to social influence**=items(19+20)/2**

Calculations of the score in biological and psychological domains, as well as the total KCS score, are performed as follows:

Biological Domain**=(A+B+C+D)/4**
Psychological Domain**=(E+F+G+H)/4**
KCS Total Score =**(Biological D.+Psychological D.)/2**

## Discussion

Research on the physical activity level in various populations or social groups, as well as actions developing indicators of such a level, are by all means valuable and necessary. To achieve an optimum activity level, a tool allowing identification of limiting factors is needed. The aim of this article was to propose such a tool – Kinesiophobia Causes Scale.

The whole spectrum of limitations of motor activity is broad. This induced the authors to assume a reductionistic attitude. The factors limiting motor activity, which were included in the scale, have been chosen on the basis of a thorough review of the literature. Our division between biological and mental factors is, however, different from the one proposed by other authors who prefer distinguishing “inner” and “outer” limitations (Daskapan et al., 2005; Gómez-López et al., 2010). In our opinion, “outer” barriers, presumably independent from the individual, would represent nothing else but mental defence mechanisms mentioned earlier in this text. Excluding rare cases of depravation of activity, it is a manifestation of individual’s will to increase or maintain the level of motor activity. In this light, our categories based on the theoretical model of health seem to aim better at the target, i.e. health prophylaxis.

It was our objective to develop possibly an universal scale serving for the purpose of identification of kinesiophobia causes in both individuals and populations. In a social dimension, the correlation of KCS score with other health status indicators (both medical based on self-assessment), in association with other important factors (e.g. culture, demographics, economy, etc.) may even constitute a base for the general health policy aiming to optimize both the level of physical activity and health status of the society. However, it should be emphasized that in case of KCS becoming widespread, modification of certain items would be necessary, taking into consideration regional and cultural issues.

On the other hand, in the field of kinesiotherapy, KCS in association with other diagnostic means applied in an individual evaluation process may become helpful in patients division, verification of indications and contraindications or indicate a need for a psychological consultation, etc.

Authors do not provide any interpretation of KCS score taking an assumption that any rigid frame of its assessment may occur debatable. Similarly to the general health status or the level of physical activity, an individual score should be regarded in the light of associated variables mentioned above. It seems that in research considering broader populations, the total KCS score (partially providing information on the level of physical activity) as well as biological/psychological domain scores may be of interest. Assessment of individual subjects should more likely go towards diagnosing of particular causes of kinesiophobia (the total or domain score seems less useful in this case). To verify the hypotheses mentioned above, broad population studies using subjects of different age, gender, culture and social status are needed.

According to the authors, the identification of causes of kinesiophobia should be followed by actions directed towards their minimization or elimination. This creates a new and broad area of research. After testing its validity, reliability and internal consistency KCS may well serve as a diagnostic tool helping to start preventive and therapeutic procedures. Authors are open to possibly broad arguments and discussions including different points of view, also those critically judging our proposition presented in this text.

## Conclusions

1. Kinesiophobia Causes Scale may constitute a useful tool for the purpose of identification and quantification of both biological and mental causes for kinesiophobic behaviours in individuals and populations.
2. From a prophylactic perspective, the identification of causes of kinesiophobia constitutes a necessary and basic start point to any coordinated actions.
